# Methods for delivering the UK's multi‐centre prison‐based naloxone‐on‐release pilot randomised trial (N‐ALIVE): Europe's largest prison‐based randomised controlled trial

**DOI:** 10.1111/dar.12592

**Published:** 2017-09-21

**Authors:** Angela Mary Meade, Sheila Macdonald Bird, John Strang, Tracey Pepple, Laura Lea Nichols, Monica Mascarenhas, Louise Choo, Mahesh Kumar Bhikhubhai Parmar

**Affiliations:** ^1^ MRC Clinical Trials Unit at University College London London UK; ^2^ MRC Biostatistics Unit University of Cambridge, Institute of Public Health Cambridge UK; ^3^ National Addiction Centre, King's College London London UK

**Keywords:** N‐ALIVE, randomised, prison‐release, naloxone, drug‐related death

## Abstract

**Introduction and Aims:**

Naloxone is an opioid antagonist used for emergency resuscitation following opioid overdose. Prisoners with a history of heroin use by injection have a high risk of drug‐related death in the first weeks after prison‐release. The N‐ALIVE trial was planned as a large prison‐based randomised controlled trial (RCT) to test the effectiveness of naloxone‐on‐release in the prevention of fatal opiate overdoses soon after release. The N‐ALIVE pilot trial was conducted to test the main trial's assumptions on recruitment of prisons and prisoners, and the logistics for ensuring that participants received their N‐ALIVE pack on release.

**Design and Methods:**

Adult prisoners who had ever injected heroin, were incarcerated for ≥7 days and were expected to be released within 3 months were eligible. Participants were randomised to receive, on liberation, a pack containing a single ‘rescue’ injection of naloxone or a control pack with no naloxone syringe. The trial was double‐blind prior to prison‐release.

**Results:**

We randomised 1685 prisoners (842 naloxone; 843 control) across 16 prisons in England. We stopped randomisation on 8 December 2014 because only one‐third of administrations of naloxone‐on‐release were to the randomised ex‐prisoner; two‐thirds were to others whom we were not tracing.

**Discussion and Conclusions:**

Prevention RCTs are seldom conducted within prisons; we demonstrated the feasibility of conducting a multi‐prison RCT to prevent fatality from opioid overdose in the outside community. We terminated the N‐ALIVE trial due to the infeasibility of individualised randomisation to naloxone‐on‐release. Large RCTs are feasible within prisons.

## Introduction

Naloxone is an opioid antagonist used by emergency services to reverse heroin/opioid overdose 1. Prisoners with a history of heroin injection have a high risk of drug‐related death (DRD) soon after prison‐release which was estimated at 5 DRDs per 1000 eligible releases on the basis of record‐linkage studies in Scotland in 1996–1999, and in England and Wales in 1999–2002 2, 3. The definitive N‐ALIVE trial was planned as a large prison‐based randomised controlled trial (RCT) to test the effectiveness of naloxone‐on‐release (NOR) in the prevention of fatal opioid overdoses soon after release. The N‐ALIVE pilot trial was a randomised feasibility study.

Internationally, a number of RCTs of public health, medicinal or nutritional interventions have been carried out in prisons. Those investigating the management of opioid addiction include an RCT in Australia in the late 1990s 4, three in the UK in the late 1990s, 2004–2005 and 2006–2008, respectively 5, 6, 7, one in Norway from 2005–2007 8 and one in the USA in the 21st Century 9. The number of prisoners randomised ranged from 382 prisoners in the Australian trial, 283 in the US trial, 46 in the Norwegian trial and 68, 90 and 306, respectively in the RCTs conducted in UK prison(s).

N‐ALIVE, however, is the largest multi‐centre, prison‐based interventional RCT in Europe and to our knowledge, including a recent systematic review by Kouyoumdjian *et al*., the largest RCT conducted in prisons worldwide 10. N‐ALIVE is also unique in that it is a trial of a prison‐based intervention which is delivered to prevent fatalities in the community.

Because the intervention in N‐ALIVE was deemed by some as contentious, by others as complex 11 and because of the prison setting, it was imperative that the highest standards of governance be adhered to throughout N‐ALIVE's conduct. On principle and in practice, participants who are prisoners should not be excluded from participating in RCTs. Prison‐based RCTs, on the other hand, must address *either* a concern that applies specifically to prisoners—to counter the challenge that the same trial could equally well have been conducted in the outside community; *or* be able to point to parallel RCTs on the outside to answer the challenge of exploiting prisoners’ captivity. Sensitive to these issues, the N‐ALIVE trial program was designed to provide a robust evidence‐base on reducing prisoners’ DRDs soon after release.

Originally, N‐ALIVE was planned as a UK‐wide study. N‐ALIVE did not proceed in Scotland because, in January 2011, Scotland became the first nation to make take‐home‐naloxone in the community, and NOR for eligible prisoners at liberation, a funded public health policy 12, 13. Wales followed suit later in 2011 14. N‐ALIVE therefore restricted its geographical scope to England where no such centrally funded public policy decision had been made.

This paper focuses on the methods of trial conduct for the N‐ALIVE pilot trial. The background, rationale and objectives for the trial and the feasibility outcome results are described separately in two publications 15, 16.

## Methods

## Trial design (planned for England and Wales; and Scotland)

**Figure 1:**
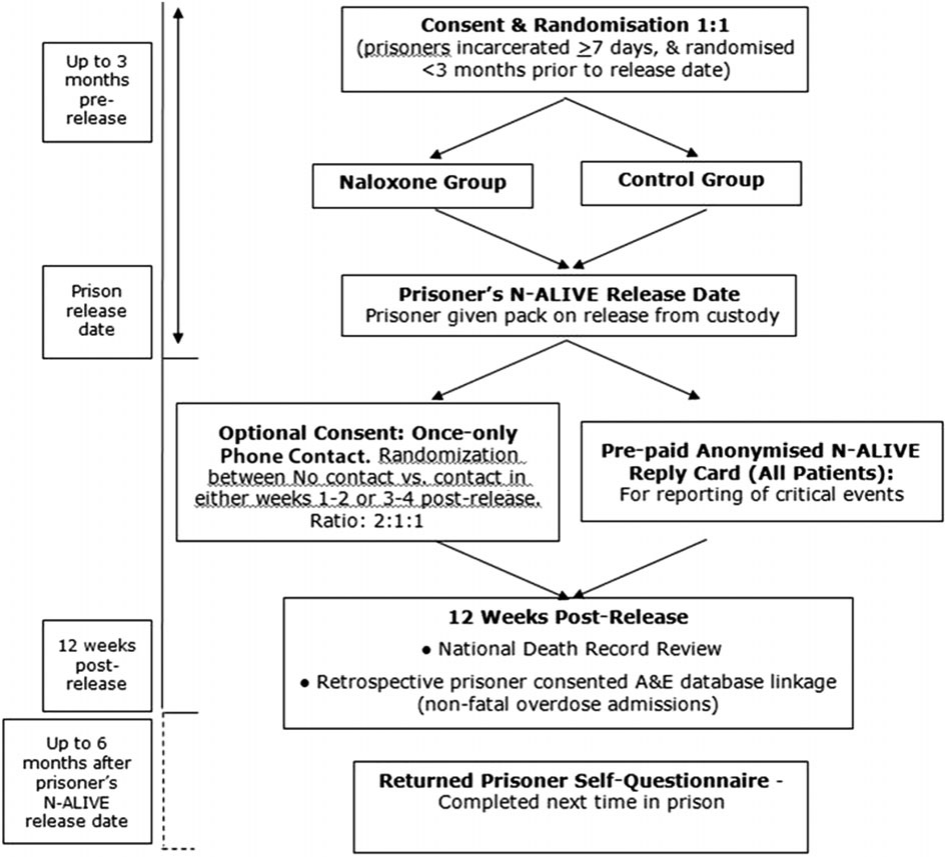
N‐ALIVE pilot trial design.

The N‐ALIVE pilot trial design, sample size calculations, data collection, eligibility, consent and randomisation processes, N‐ALIVE packs, returned prisoner self‐questionnaire, primary and secondary outcomes and statistical analysis plan are described in Parmar *et al*. 16 and in the N‐ALIVE protocol published on the Medical Research Council (MRC) Clinical Trials Unit (CTU) website 17. The trial design is summarised in Figure 1. In brief, eligible consenting participants were randomised (1:1) to receive, at liberation, a pack containing either a single ‘rescue’ injection of naloxone (an opioid agonist) or a control pack which did not contain naloxone. The trial was double‐blind up until the point of release so that neither the participant nor prison‐based N‐ALIVE staff nor prison staff knew the allocation while the participant was in custody. Participants learned their allocation when they opened the pack immediately after their release from prison.

The trial included a randomised sub‐study in which participants who gave consent for once‐only telephone contact after release were randomised further between no contact and telephone contact in the first versus second fortnight after release in the ratio of 2:1:1. The rationale for the 50% no‐contact‐rate was to minimise contamination of the main randomisation as the telephone‐interview asked participants, inter alia, if they were carrying naloxone—in effect, reminding them to do so.

In addition, an optional consent enabled former N‐ALIVE participants who returned to prison within 6 months of their most recent N‐ALIVE release to be invited to complete a Returned Prisoner Self‐Questionnaire (RPSQ): 85% of randomised participants consented (1417 of 1676). Using the RPSQ, we collected information about naloxone carriage, its administration and heroin use after release from prison 16.

### User and service engagement

During the trial design stage, the trial's co‐principal investigators (PI) met with prisoners with a history of heroin injection. A number of prisoners took part at each of two prisons, one in Scotland and one in England. Prisoners’ feedback informed planning of the trial's logistics and conduct. For example, a prisoner illustrated to the PIs the size of a small tin that, on liberation, would contain all of his valuable possessions (which were few). This led to a desire for a naloxone kit which would be no larger than the size of a credit card which led in turn to the decision to contain the naloxone kit within a wallet. Organisations and others who work with heroin users, such as SPODA (an organisation supporting families and carers of drug misusers) and families against drugs also contributed to the trial design, including the N‐ALIVE DVD 18, 19.

## Trial set‐up (England only)

### National approvals

**Figure 2:**
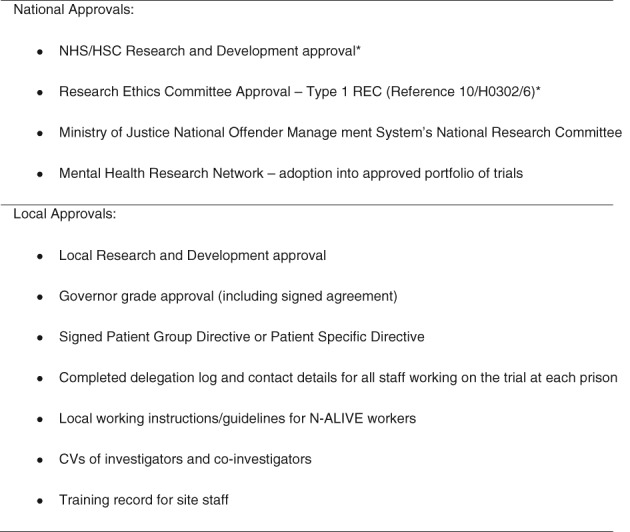
National and local approvals for N‐ALIVE**.** *If the N‐ALIVE pilot trial were being conducted today we would apply for approvals through the Health Research Authority process. HSC, Health and Social Care; NHS, National Health Service.

Figure 2 lists the many approvals that had to be obtained in order to get N‐ALIVE pilot trial up and running.

### Links with the mental health research network

The English Mental Health Research Network (MHRN) adopted the N‐ALIVE pilot trial into its portfolio of studies, and thus supported us in setting the trial up at prison sites and provided clinical studies officers to perform the role of prison‐based N‐ALIVE worker at many of our prisons.

### Prison selection and local approvals

We approached 20 large prisons holding prisoners of security category B (no need for high security conditions) and C (for prisoners not trusted with open conditions, but unlikely to make a determined escape attempt). MHRN co‐ordinators also suggested prisons in their network area that might be suitable.

We began visiting prisons in February 2011. The trial was designed to cleave to prison procedures, yet within each prison we encountered different methods of operation. It took 13 months to get approval from the first prison and a further 3 months before they opened to participant accrual. Reasons for delays in prison set‐up included the lengthy process to agree, obtain and issue research and development approval, appointing N‐ALIVE workers (often delayed for funding reasons) and obtaining their security clearance, agreeing the pack prescription method and completing and collating trial accreditation documents.

**Figure 3:**
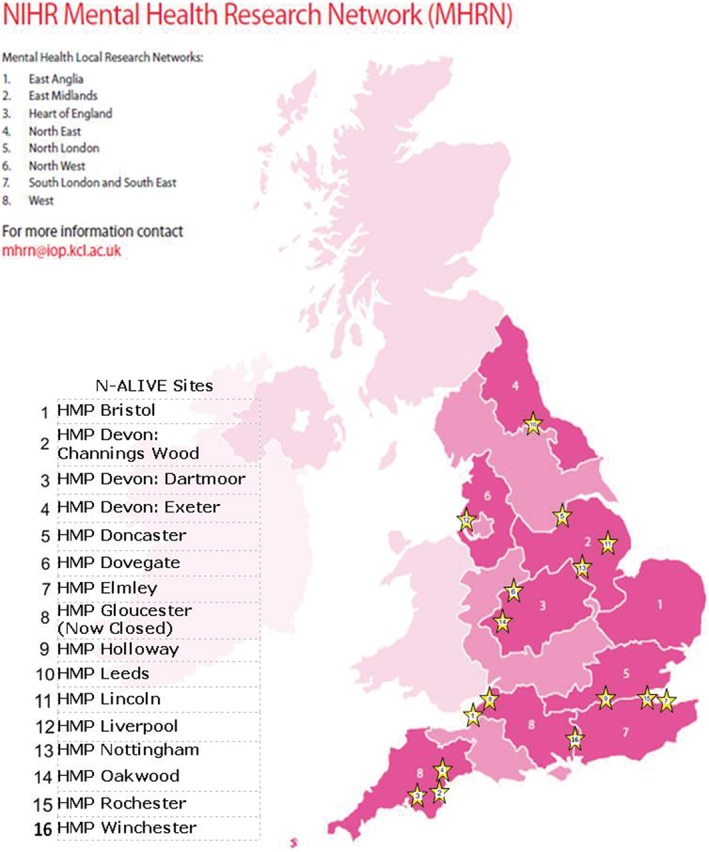
Map of N‐ALIVE prisons and the Mental Health Research Network hubs.

In most cases, the preferred first step with each prison was a meeting at the prison with as many stakeholders as possible (a person of Governor grade, security and health‐care representatives, and, if possible, a candidate for the N‐ALIVE worker role). During these meetings, the rationale for the trial was presented and details of how the trial might be conducted were introduced; questions about the trial were answered and any concerns discussed. A PI was identified to lead the research team at each prison. See Figure 3 for a map of N‐ALIVE prisons and MHRN hubs.

We were unsuccessful in our attempts to engage with a number of prisons because of security concerns, workload issues and even prison organisational changes, for example, re‐rolling of prison category or possibility of closure or privatisation. At one prison, short‐term funding via National Health Service (NHS) England's ‘Through the prison gateway’ scheme led to their own non‐randomised take‐home‐naloxone program shortly after they had been approved to participate in the N‐ALIVE pilot trial.

### N‐ALIVE DVD

We produced an instructional film on DVD which we also posted on YouTube and on the trial web‐page 19. The film had a three‐part structure: (i) ‘The N‐ALIVE Heroin Overdose Study’; (ii) ‘What's in the Pack?’; and (iii) ‘Giving the Naloxone’. The DVD was provided to all participants in their N‐ALIVE pack (both naloxone and control) and they were encouraged to share its viewing with family and friends.

### The N‐ALIVE worker role

A key role in each prison was that of the N‐ALIVE worker. Their responsibilities included identifying potentially eligible participants, explaining the trial, taking consent, randomising, completing case report forms and ensuring that participants received their packs on release; all the while maintaining close links with MRC CTU and with prison staff. In some prisons, the N‐ALIVE worker role was undertaken by the MHRN clinical studies officers and/or staff seconded from other health‐care roles in the prison. These posts were funded by the MHRN or by the Comprehensive Local Research Network in which the prison was located.

We provided trial‐specific training to N‐ALIVE workers, usually face‐to‐face. Once one person at a prison site received training, he/she could train other colleagues at that prison. N‐ALIVE workers also completed Good Clinical Practice training and were advised to watch the N‐ALIVE DVD.

### Prison‐specific training

N‐ALIVE workers had to have hepatitis B immunisation, Criminal Record Bureau security clearance and undertook key training before they could have access to the prison.

### N‐ALIVE packs: choice of naloxone product and description of other contents

The N‐ALIVE co‐PIs agreed that naloxone should be provided as an intramuscular injection (which does not require the specialist skills required for intravenous injection) and preferably in a syringe pre‐loaded with naloxone (and therefore ready to be administered without any further preparation).

**Figure 4:**
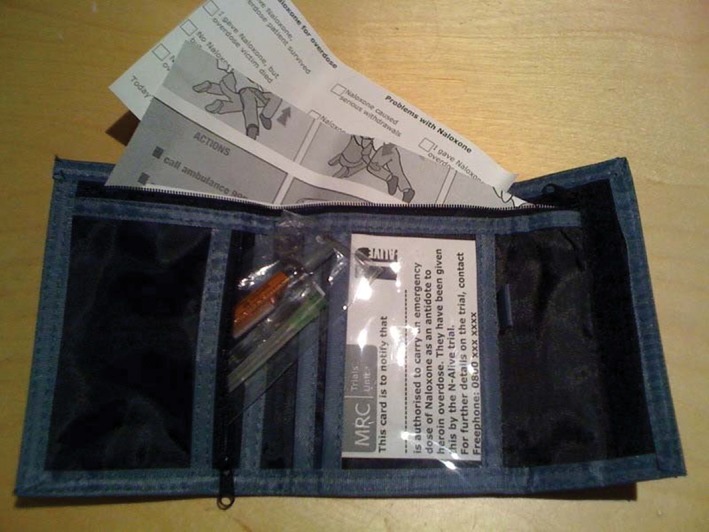
The N‐ALIVE naloxone wallet. N‐ALIVE trial wallet containing the naloxone‐filled syringe and needle, information on overdose management and ‘authorisation to carry’ card.

Three pharmaceutical formulations of naloxone existed on the UK market at the time of trial planning: first, a traditional glass ampoule of naloxone 0.4 mg; second a pre‐filled special Mini‐Jet syringe of 0.4 mg and third a pre‐filled standard syringe of 2 mg. None of these formulations contained the correct identified dose of 0.8 mg, and none had a stake needle (moulded/fixed needle). It became clear that a stake‐needle variant would only be available to the N‐ALIVE trial team as a ‘special’ preparation which would render the medication an investigational medicinal product which would have additional implications for trial conduct. The option of giving the N‐ALIVE participant two 0.4 mg ampoules or mini‐jets was ruled out because of the risk of contamination between the intervention and control groups; a participant leaving prison with two ampoules or mini‐jets could give one of them to a fellow released prisoner who had been assigned to the control group. Thus, the naloxone needed to be a single product for the purpose of the trial. None of the available products fitted our needs perfectly, but we concluded that a sufficient dose in single product form was most crucial. Accordingly, we selected the 2 mg pre‐loaded syringe as an acceptable formulation, in which there is more than adequate dose. This choice of dose necessitated additional instructions for administration to be limited to a 0.8 mg dose. We therefore developed instructions, and the DVD and YouTube contributions to teach the administration of a 0.8 mg dose from the 2 mg pre‐filled syringe formulation and safe disposal of the syringe to avoid its reuse for another person. The method of naloxone delivery, including the choice of dose is also described in Strang *et al*. 15. The N‐ALIVE packs are described in Parmar *et al*. 16; a picture of the naloxone wallet is shown in Figure 4.

We entered into correspondence with the UK Medicines and Healthcare Regulatory Agency to confirm that the trial was not a clinical trial of an investigational medicinal product. The Medicines and Healthcare Regulatory Agency accepted that the outcomes of the pilot trial were not related to the drug efficacy, safety, metabolism or pharmacodynamics, or indeed, to the drug in anyway, but concerned the treatment management strategy only. Naloxone was being used within its license and we would not be gaining any further information about the authorised form.

The rationale for providing both groups with a sealed N‐ALIVE pack (box) containing the wallet at the point of prison‐release was in order to preserve the double‐blind assignment up until the point of liberation. This safeguarded prisoners from undue pressure from peers while in custody; and ensured that N‐ALIVE workers could not subconsciously or deliberately support or add precautionary measures or behave differently towards those prisoners who had been assigned to the control group. A common standard of care‐as‐usual therefore applied until the point of release when the ex‐prisoners were given their allocated N‐ALIVE pack and opened it to discover their allocation.

### Getting packs to prisons

Once a prison was approved to participate in the pilot trial, a stock of pre‐numbered N‐ALIVE packs was issued to it. N‐ALIVE packs were stored securely within prison pharmacies or in an alternative secure location. We provided lockable cabinets to prisons on request.

### Prescribing naloxone

In consultation with their local NHS trust, research teams at the prisons chose one of two procedures for prescribing naloxone to participants**:** the patient specific direction (PSD) or the patient group direction (PGD). A PGD is a legal document which allows registered doctors, nurses or pharmacists to prescribe a product without the need for individual prescriptions so long as they have received training on the PGD. Once a PGD has been approved there is no additional paperwork to complete for individual participants. A PSD is a written instruction from a qualified, registered prescriber for a medicine, including the dose, route and frequency to be supplied to a named patient. A PSD was generally used when the N‐ALIVE worker was not a qualified prescriber. A separate entry was made on the PSD form and signed off by the local PI prior to randomisation of each participant. Because the trial was double‐blind until the participants opened their pack on release, the same procedures were followed for all participants whilst in prison. Consequently all packs were documented on the PGD/PSD irrespective of whether they contained naloxone or control, as staff would have been unaware of pack contents.

### Trial oversight

A Trial Management Group (TMG) was formed by the three co‐PIs and the MRC CTU trial team who were responsible for the day‐to‐day management of the trial. The TMG met (usually monthly) either in person or via teleconference, or discussed issues via email as appropriate. The very broad remit of the TMG was to manage the trial, including the clinical and practical aspects.

We established a joint Trial Steering and Data Monitoring Committee (TS‐DMC); the committee paid particular regard to accrual and changes in national policy which might impact on the continuing need for the N‐ALIVE main trial. The latter was important because we had given an undertaking to participants and to the Research Ethics Committee (REC) that recruitment to the N‐ALIVE pilot trial would cease if we had reason to know that the main trial could not go ahead. The committee met on five occasions during the feasibility trial. We developed Charters for both the TMG and TS‐DMC which were agreed and signed by all members.

### Confidentiality

With their explicit consent, participants’ name, date of birth, sex and NHS number were disclosed to us to enable database linkages to inform the trial's outcome measures. Participants’ names were stored separately from the other information held about them.

All consent forms and copies of case report forms were stored securely within the prison. Copies of the consent form were sent to MRC CTU to be checked and were then destroyed. To encourage frankness, completed RPSQs identified only the participant's treatment assignment in N‐ALIVE pilot trial, the month and year of their most recent N‐ALIVE release, and the month and year of RPSQ completion.

Participants who consented to the telephone contact sub‐study were asked to provide up to two telephone contact numbers: 56% of participants consented to the sub‐study (946 out of 1676 randomised); 95% confidence interval 54%–59%. The telephone numbers were stored in separate secure databases. At the initiation of the telephone call, we did not disclose that the participant was an ex‐prisoner or that N‐ALIVE aimed to reduce DRDs. Eighty‐one interviews were successfully conducted.

### Systems and databases at MRC CTU at UCL

We used an in‐house randomisation system which we linked to our database inventory of N‐ALIVE packs. When unassigned packs at a prison reached a certain threshold, an email was generated and used to inform the pack distribution company about which additional trial packs to supply to that prison.

The database for managing the clinical trial data was developed and managed in‐house using MACRO version 4. A separate database was developed as a repository for data from the RPSQ and telephone questionnaires.

## Trial conduct during recruitment

### Identifying potential participants

N‐ALIVE workers liaised with members of the health care/addictions staff at their prison to find the best approach for identifying potential participants. Self‐referral was encouraged and posters advertising the trial were provided for display in the wings, health care, methadone‐dispensary, reception and visiting areas. Word‐of‐mouth communication between prisoners about the trial was widely reported by our N‐ALIVE workers.

### Participant information and informed consent

Our prior logistical plan was that potentially eligible prisoners would learn about the trial in group sessions for six to eight prisoners who could then request a one‐to‐one individual consent session. In practice, group sessions were not feasible in most prisons due to unavailability of private space or restrictions on prisoner movement.

We produced a summary and a full patient information sheet. The summary patient information sheet could be provided when prisoners initially expressed interest in the trial. The full patient information sheet could also be given at this point or held in reserve until the potential participant had time to digest the initial information. Potential participants could take as long as they needed to consider whether or not to take part in the trial. If the N‐ALIVE worker had any concerns about a prisoner's comprehension of the trial, he/she encouraged to discuss his/her concerns with a member of the health‐care team prior to obtaining consent.

### Randomisation

To randomise a participant, the N‐ALIVE worker contacted the MRC CTU by telephone. Once the patient's eligibility was confirmed, the N‐ALIVE worker was provided with the participant's pack number. This pack number was the participant's unique identifier in the trial (‘trial number’). The randomisation method (80:20 minimisation) is described in Parmar *et al*. 16.

### Shortfall in accrual

**Figure 5:**
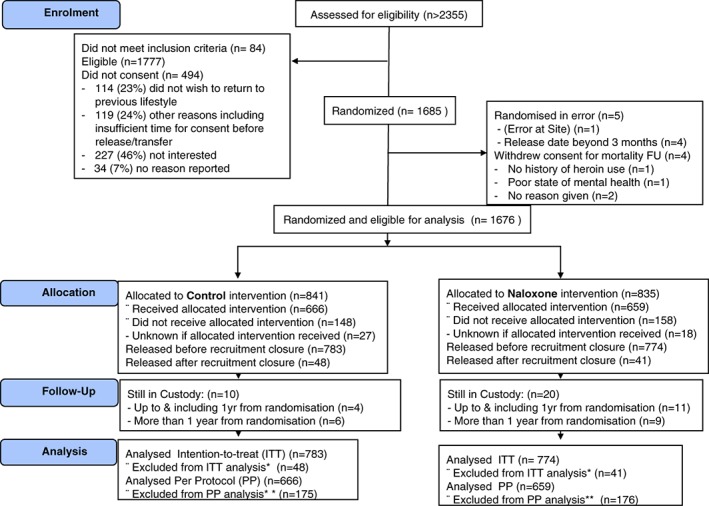
CONSORT diagram (originally published in Parmar *et al*. [16]). Screening records have only been kept since September 2012 so only provide a snapshot of the proportions deemed eligible and subsequently randomised. *Excluded from ITT analysis participants released after recruitment closure (*n* = 48, 40). **Included in PP analysis participants released with pack only.

Despite being the largest European prison‐based interventional trial to date, we did not meet our accrual target of 2800 prisoners from English prisons. The original protocol target was up to 10% of the number of patients required for the main trial; the target was reduced to 2800 when the N‐ALIVE pilot trial could randomise in English prisons only. The justification for the original sample size is described in Parmar *et al*. 16. Between 28 May 2012 and 8 December 2014, we randomised 1685 participants (842 to naloxone, 843 to control) 16 (Figure 5). The reticence to randomise remand prisoners (who have court appearances to make) was a factor in reduced accrual, as was insufficient N‐ALIVE worker time at prisons. Other reasons included: slower than anticipated set‐up time for new prisons, fewer prisoners with a history of heroin injection, outdated prior estimates based on prisons’ drug treatment clients in 2005 and some potential participants who, because they wanted to move away from opiate use, worried that carrying naloxone might be interpreted by family and friends as a lack of resolution.

Slower than anticipated accrual meant that the stock of naloxone we ordered at the start of the trial expired before it could be used and consequently we needed to purchase new stock and replace the soon to be expired stock. Both naloxone and control N‐ALIVE packs at sites were replaced to preserve the integrity of the blind allocation.

### Participant data

N‐ALIVE workers provided data on paper case report forms; completed case report forms were sent to MRC CTU at UCL via fax or post.

### Storing and getting packs to participants on release

Prisoners routinely receive their valuable property and/or medication as they are released from prison or from court and these valuables are transferred with them if they change prison. In theory, N‐ALIVE would cleave to this process, and once allocated by randomisation, the participant's N‐ALIVE pack would be placed with his/her valuable property. In practice, storage of the N‐ALIVE pack with the prisoner's valuable property was not always possible for a variety of reasons generally relating to security concerns. Trustee prisoners in some prisons had a degree of access to valuable property (and half the N‐ALIVE packs contained a needle) while, in other prisons, there were concerns that prisoners would get access to a needle before they had properly exited the prison on the day of their release. Another concern was a misconception that provision of an N‐ALIVE pack by security staff to a participant on their release might constitute secondary prescribing. As prisons gained experience, increasing numbers of prisons allowed packs to be placed in prisoner's valuable property.

N‐ALIVE workers worked around the embargo on placing packs in valuable property and made alternative arrangements for prisoners whom they were expecting to be released or transferred. However, prisoners are often released, returned to court or transferred at very short notice. If a participant was transferred to another N‐ALIVE prison, then the trial team or the N‐ALIVE worker could often arrange transfer of the prisoner's allocated pack retrospectively, but this was much more difficult if participants were transferred to non‐N‐ALIVE prisons as we did not have the contacts to facilitate pack transfer. There were exceptions, staff at a number of non‐N‐ALIVE prisons accepted N‐ALIVE packs as participant prisoners’ property. Unfortunately, even after successful transfer to non‐N‐ALIVE prisons, we were often unable to confirm whether participants had received their packs when they were eventually released.

### Obtaining personal outcome data from national registries

Formal approval was obtained from the Office for National Statistics for the provision of mortality data. We obtained matched mortality data on N‐ALIVE trial participants using a secure data transfer process. Results are described in Parmar *et al*. 16.

### Communicating with N‐ALIVE workers, prisons and prisoners

To ensure timely answering of queries from N‐ALIVE workers, we set‐up a trial team email address. We also set‐up an N‐ALIVE worker email group and encouraged N‐ALIVE workers to communicate directly with each other. Together with the N‐ALIVE workers, we engaged with as many members of prison staff as we could. In addition to initial training for each site, we held a well‐received N‐ALIVE worker and PI meeting in June 2014 where everyone shared experiences and best practices. We produced regular newsletters and we presented trial updates at annual MHRN conferences. N‐ALIVE was also featured on prison radio and in Inside Times, the newspaper for prisoners.

## Trial close‐out

### Early cessation of randomisation

An interim review of the N‐ALIVE pilot trials feasibility outcomes was undertaken to coincide with the release of official statistics on the third year of Scotland's National Naloxone Programme 21. Examination of the responses to the RPSQs identified that only a third of the emergency naloxone administrations to reverse overdose were to the randomised ex‐prisoner, two‐thirds were to an individual who was not the study subject 16. Our data capture methods were not able to identify, let alone consent, these other individuals. This finding, which was backed up by the Scottish results 22, had major implications for the main N‐ALIVE trial, which was designed around individual randomisation. Consequently, the TS‐DMC unanimously agreed that randomisation to the N‐ALIVE pilot trial should cease on 8 December 2014 20. This decision was also approved by our appointed REC.

Our TS‐DMC also proposed (and the REC‐approved) that, with the approval of the PIs at each prison, participants who remained in custody on the morning after accrual stopped should be offered naloxone‐on‐release, irrespective of their randomised allocation 20. We produced a letter for trial participants still in custody to explain these changes; this was also REC‐approved prior to circulation. We updated our N‐ALIVE web‐page with details of all decisions 23.

We communicated both the unplanned closure and the plan for participants remaining in custody to our N‐ALIVE workers and local PIs by email together with the reasoning behind the decisions; followed by details on how to manage the changes.

### Final steps at sites

From 19 June 2015, N‐ALIVE packs were no longer provided to participants. All participants were expected to have been released within 3 months of their randomisation; the 19 June cut‐off date allowed for another 3 months’ grace. Other factors that influenced this decision included the expiry date on the naloxone packs and the fact that many of our N‐ALIVE workers were no longer regularly available. Further data collection from sites also stopped on this date in compliance with our protocol (6 months after accrual of the last participant). Both of these decisions were approved by our REC and then relayed to our N‐ALIVE workers for execution 24. N‐ALIVE workers also helped prepare sites for overall trial closure. Some sites were unable to archive the trial files for the required 20 years; files were thus transferred to UCL for storage.

## Discussion

We have unequivocally demonstrated the feasibility of conducting a large randomised trial in multiple prisons. The approval and conduct of the N‐ALIVE trial involved multiple agencies and policy environments, many of which were subject to considerable change in the course even of the N‐ALIVE pilot trial. Support of the MHRN was invaluable.

Good communication has been essential. We needed key members of staff at the prison (Governor, health care, reception and security staff) to be aware of the trial and the trial processes. Most prisons were very supportive of N‐ALIVE and this support generally increased as prisons gained experience with the trial. Some prisons were less engaged, but usually this was because they had competing concerns which took priority over N‐ALIVE. The Governors at our N‐ALIVE prisons understood our objectives and were willing to work with us; their main concern being the safety, security and wellbeing of all their inmates and staff in what can be a very hectic and challenging environment. Clinical trials are carried out to high standards following recognised guidelines and procedures; this was reassuring for prisons where rule and order are valued. At one prison, our N‐ALIVE worker was experiencing some difficulty getting security staff to help with getting packs to participants on their release. The Governor engaged with us to document and approve a policy that became part of routine procedure in that prison. We then shared this policy with other prisons.

Prisoner approval for the trial was high 16. N‐ALIVE workers reported enthusiasm for the idea behind the trial even amongst those who decided not to participate (such as prisoners who had undergone detoxification and wished to abandon their former lifestyle). One of our prisons reported that participants had asked about having a certificate to say that they had attended the information groups and were participating in a trial as they felt that this would be favourably perceived by probation and the courts. Feedback received from participants during the follow‐up telephone calls and in the additional comments section of the RPSQ was generally positive, insightful and supportive 16. It was the responses to the questions about what the participants did with their naloxone that showed us that individual randomisation was not appropriate for the main trial and led to the decision to cease randomising to the pilot trial.

Delayed registration of deaths in England means research teams like ours who seek to verify the survival‐status of study‐participants are informed not about deaths which have occurred, but about deaths which have occurred and have been registered as having occurred. The two differ importantly because the delay to allow cause of death to be established by inquest and then registered can be one year or more. For us, this meant we had to wait to report on the number of DRDs among our N‐ALIVE participants. Similar delays will be experienced by others until legislation is introduced to uncouple registration of the fact of death from registration of the cause of death. This is something Professor Bird and the UK Royal Statistical Society have argued for over the last many years 25, 26, but requires legislation which—to date—has not been brought forward.

Furthermore, the introduction of a unique prisoner number system across the entire prison population greatly facilitates research in general. We benefitted from the unique prisoner numbering system which enabled the majority of N‐ALIVE prisoners to be re‐contacted on re‐incarceration. We now need to see this number system being used across all prisons—private as well as public.

## Conclusions

We have shown that it is possible to successfully conduct large‐scale randomised trials across multiple prisons. We hope that others will use the N‐ALIVE pilot trial experience as a basis for undertaking more trials in prisons and other complex environments.

## Conflict of Interest

AMM, TP, LLN, MM, LC and MKBP have no conflicts of interest. JS is a researcher and clinician who has worked with a range of types of treatment and rehabilitation service‐providers, including treatments within prison and on prison‐release. JS is supported by the National Institute for Health Research Biomedical Research Centre for Mental Health at South London and Maudsley NHS Foundation Trust and King's College London. He has also worked with a range of governmental and non‐governmental organisations, and with pharmaceutical companies to seek to identify new or improved treatments (including naloxone products) from whom he and his employer (King's College London) have received honoraria, travel costs and/or consultancy payments. This includes work with, during the past 3 years, Martindale, Reckitt‐Benckiser/Indivior, MundiPharma, Braeburn/MedPace and trial medication supply from iGen. His employer (King's College London) has registered intellectual property on a novel buccal naloxone formulation with which JS is involved. JS has also been named in a patent registration by a Pharma company as inventor of a concentrated nasal naloxone spray. For a fuller account, see JS's web‐page at http://www.kcl.ac.uk/ioppn/depts addictions/people/hod.aspx. SMB served on Scotland's National Naloxone Advisory Group and co‐authored the peer‐review paper on before/after evaluation at 3 years of Scotland's National Naloxone Policy. SMB holds GlaxoSmithKline shares.
